# Effectiveness of Respiratory Muscle Training in Pompe Disease: A Systematic Review and Meta-Analysis

**DOI:** 10.3390/children11101209

**Published:** 2024-09-30

**Authors:** Mu-Yun Lin, Szu-Han Chen, Jen-Ting Lee, Po-Cheng Hsu

**Affiliations:** 1 Department of Physical Medicine and Rehabilitation, Taipei Veterans General Hospital, Taipei City 112201, Taiwan; mylin16@vghtpe.gov.tw (M.-Y.L.); jtlee2@vghtpe.gov.tw (J.-T.L.); 2 School of Medicine, College of Medicine, National Yang Ming Chiao Tung University, Taipei City 11221, Taiwan; 3 Department of Physical Medicine and Rehabilitation, West Garden Hospital, Taipei City 108035, Taiwan; 4 Department of Physical Medicine and Rehabilitation, National Taiwan University Hospital, Bei-Hu Branch, Taipei City 10845, Taiwan

**Keywords:** Pompe disease, respiratory muscle training, systemic review, meta-analysis

## Abstract

**Background**: Pompe disease is a rare metabolic myopathy caused by the lack or deficiency of the lysosomal acid alpha-glucosidase, resulting in skeletal muscle weakness and cardiomyopathy. The disease varies by onset age and genetic mutations and is categorized into infantile-onset and late-onset Pompe disease. Respiratory muscle weakness may persist regardless enzyme replacement therapy. This systemic review and meta-analysis aim to assess the effect of respiratory muscle training (RMT) on respiratory muscle strength, functional endurance, and pulmonary function in patient with Pompe disease. **Methods**: PubMed, EMBASE, and Cochrane databases were searched up until Aug 2024. Studies examining the therapeutic effects of RMT in patients with Pompe disease were included. Outcome measures included the change in maximal inspiratory pressure (MIP), maximal expiratory pressure (MEP), six-minute walking test (6MWT), pulmonary function before after RMT, quality of life and adverse events. **Results**: The meta-analysis consisted of 5 single-arm studies, including 31 patients in total. Regarding inspiratory muscle strength, RMT has significantly improving MIP (8.71 cmH_2_O; 95% CI, 6.23–11.19, *p* < 0.001) and MEP (12.15 cmH_2_O; 95% CI, 10.55–13.74, *p* < 0.001) in both types of Pompe disease. However, no significant change regarding 6MWT. No serious adverse events were reported. **Conclusions**: Our meta-analysis revealed that RMT may increase inspiratory muscle and expiratory muscle strength, but may not have an effect on 6MWT in patients with Pompe disease. RMT has potential to be integrated into the cardioplulmonary rehabilitation for patients with Pompe disease. Further large randomized controlled trials are needed to verify the efficacy and safety of RMT in patients with Pompe disease.

## 1. Introduction

Pompe disease, known as glycogen storage disorder type II or acid maltase deficiency, is a rare metabolic muscle disorder that passed down through autosomal recessive inheritance. A deficiency or lack in the lysosomal enzyme acid alpha-glucosidase (GAA) results in the buildup of glycogen in the smooth, cardiac, and skeletal myocytes, which causes weakness of the affected muscles, often resulting in premature death [1]. The incidence of Pompe disease differs across ethnicities and regions, and it affects approximately 1 in 40,000 to 1 in 200,000 individuals globally [2,3,4]. The clinical manifestation of Pompe disease varies based on factors such as the age of onset, specific genetic mutations involved, and rate of disease progression [5]. Pompe disease is classified into two forms according to the residual GAA activity: late-onset Pompe disease (LOPD) and infantile-onset Pompe disease (IOPD) [6].

IOPD arises from a complete or near-total absence of GAA activity. Symptoms of IOPD typically manifest within the first few days to weeks after birth and include respiratory insufficiency, hypertrophic cardiomyopathy, reduced muscle tone and severe muscle weakness [7]. Without adequate treatment, fatal cardiorespiratory failure can occur by the age of 24 months [8].

Treatment options for respiratory muscle weakness caused by Pompe disease are limited. Since its introduction in 2006, the enzyme replacement therapy (ERT) with alglucosidase alfa (Myozyme™) has significantly enhanced both overall and ventilator-free survival in children with IOPD [9,10,11]. However, in few children with IOPD, respiratory muscle weakness can persist despite ERT. This weakness can lead to hypoventilation, diminished cough effectiveness, and a reduced capacity for physical activity [12,13,14].

The first RMT study on IOPD patients by Jones et al. demonstrated the effectiveness and safety of respiratory muscle training (RMT) for improving the maximal expiratory pressure (MEP) and maximal inspiratory pressure (MIP) in IOPD patients [14]. Moreover, the findings were supported by the replicated study, and proved that these effects are maintained after detraining [15].

Patients with LOPD typically have a more insidious course and experience proximal muscle weakness that becomes evident around the age of 30 years [16]. Respiratory muscle weakness is frequently noticed while diagnosis, in which the inspiratory muscles are primarily affected [17], and respiratory-muscle weakness leads to reduced airway clearance, impaired cough [18,19], sleep-disordered breathing [20,21], progressive respiratory insufficiency [22], and acute or chronic respiratory failure [23]. Notably, respiratory functional outcomes of ERT are inconsistent in patients with LOPD. Few studies have reported a notable deceleration in LOPD progression with stabilization of pulmonary function [24,25,26]. However, a prospective study that recruited 177 patients did not demonstrate a similar effect on respiratory support [27,28]. Moreover, extended observational studies have revealed that approximately one-third of the patients exhibit an inadequate response to treatment and experience a decline in pulmonary function, eventually requiring ventilator support [29,30]. Therefore, additional interventions, such as RMT, are important to enhance the cardiopulmonary capacity in individuals with Pompe disease [31,32,33,34]. Martin et al. reported that inspiratory muscle training (IMT) improved lung function, muscular strength, and endurance in a patient with LOPD [35]. Furthermore, three trials assessing the effects of IMT in LOPD patients reported a significant improvement in respiratory muscle strength, but not in pulmonary function, after IMT [34,36,37].

Recently, Bordoli et al. published the first systematic review on the efficacy of physical training in glycogen storage disorders [38]. They included studies on patients with McArdles and Pompe diseases, and seven studies focused on Pompe disease. Improvements in respiratory muscle strength and additional benefits, such as improvements in the aerobic fitness, functional endurance, and general health, were observed.

While ERT remains a cornerstone treatment, its impact on respiratory dysfunction is inconsistent. Additional RMT is considered important for patients with Pompe disease, by enhancing pulmonary outcomes and improving functional capacity and quality of life for patients. No previous meta-analysis has systematically explored the effects of RMT on respiratory muscle strength, pulmonary function, and functional endurance in this population. Therefore, our systematic review and meta-analysis aimed to assess these effects and provide potential clinical hint in improving comprehensiveness of Pompe disease care.

## 2. Materials and Methods

### 2.1. Search Strategy and Criteria

The MEDLINE (PubMed), Cochrane databases, and EMBASE were searched for eligible articles. The search was performed in August 2024, utilizing the terms “Pompe disease” and “respiratory muscle training” either individually or in combination, along with a specific filter for related studies. No restrictions were applied regarding the language or year of publication. The detailed search strategy used for PubMed is shown in the Supplementary Data S1. This meta-analysis was not carried out following any publicly available or registered protocols.

### 2.2. Inclusion and Exclusion Criteria

The inclusion criteria were defined as follows: (1) studies design of randomized controlled trial, non-randomized trial, or pilot study (2) enrollment of patients with Pompe disease, regardless of age, including both IOPD and LOPD. (3) implemented either an expiratory (EMT) or IMT protocol, irrespective of the device type used. No limitations were placed on the dosage, timing, setting, or level of supervision for the intervention. However, studies involving additional interventions, such as medication or other elements of pulmonary rehabilitation, were excluded unless these interventions were uniformly applied across all participant groups. The outcomes were as follows: pulmonary function, functional endurance (6-min walk test [6MWT]), respiratory muscle strength, peak cough flow, and quality of life (QoL). Studies were excluded if they were case reports or if they lacked sufficient statistical data for inclusion in the meta-analysis.

### 2.3. Study Selection

Two reviewers (M.-Y.L. and P.-C.H.) independently assessed the identified titles and abstracts. If the abstract had insufficient information regarding the inclusion and exclusion criteria, the full text was assessed for eligibility according to the inclusion criteria.

### 2.4. Data Collection and Extraction

Two authors collected data independently using a pre-designated evaluation form. The following data were gathered: (1) demographic and clinical characteristics of the study population, including age and types of Pompe disease; (2) intervention parameters inlcuding duration, intensity, detailed training protocol, and frequency; (3) primary outcome including MIP and MEP; (4) Secondary outcomes including functional capacity (evaluated using the 6MWT), pulmonary function (evaluated using peak cough flow, forced vital capacity [FVC], and forced expiratory volume in 1 s (FEV1)), and QoL.

### 2.5. Assessment of Study Quality

The methodological quality, evaluated by NIH quality assessment tool for before-after (pre-post) studies, of the enrolled studies was independently by two authors (M.-Y.L. and P.-C.H.) [39]. All quality assessment tools were modified to align with the specific requirements of this review. Certain criteria were marked as” not applicable”, and the total score was adjusted accordingly. For the quality of case-control studies, a score between 7 and 12, between 5 and 6, and less than 5 was defined as “good”, “fair”, and “poor”, respectively.

### 2.6. Data Synthesis and Analysis

We applied the random-effects model to pool the effect sizes owing to variability in study design (such as training intensity, frequency, and medications used) across the included studies. The effect measures were calculated using the difference between the means and standard deviations (SDs) of the means. For studies that did not provide the SDs of the changes, we imputed them using the formula from the Cochrane Handbook for Systematic Reviews of Interventions. This formula is presented as Supplementary Data S2. The 95% confidence interval (CI) was considered significant. We conducted a subgroup analysis of LOPD. I-square values of 25%, 50%, and 75% implied low, moderate, and high heterogeneity, respectively. Potential publication bias was assessed using the Egger’s regression test and symmetry of the effect-size distribution on a funnel plot. All analyses were performed using STATA 14 (version 14.0; Stata Corp, College Station, TX, USA).

## 3. Results

### 3.1. Study Identification and Selection

**Figure 1:**
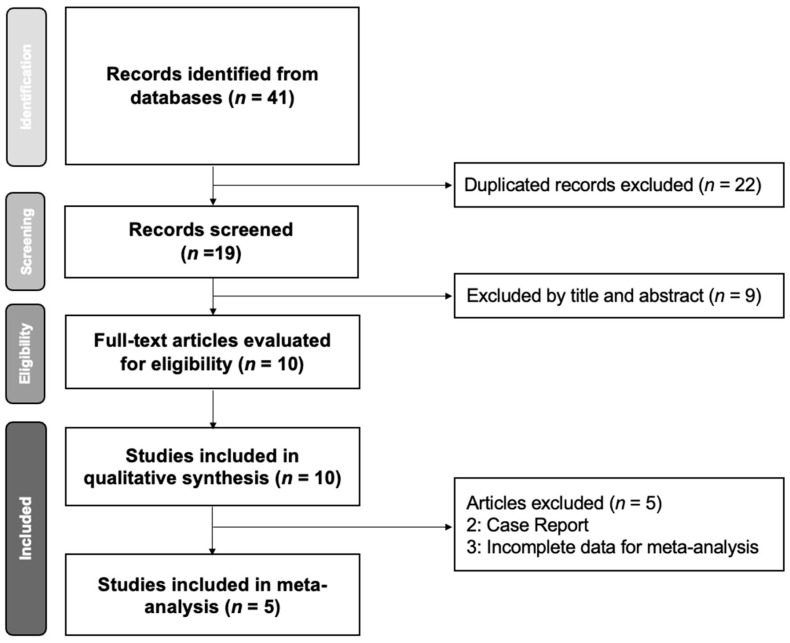
Preferred Reporting Items for Systematic Reviews and Meta-Analyses (PRISMA) flow diagram for the study selection process.

The initial search retrieved 41 studies. After removing 22 duplicate articles and excluding 9 non-relevant articles based on title and abstract screening, 10 studies were considered eligible for further evaluation. Three and seven studies focused on IOPD [14,15,40] and LOPD, respectively. However, only one study included a control group and an intervention group [15]; therefore, a single-arm meta-analysis was preferred to evaluate the therapeutic effects. Among the 10 studies, six were included in the single-arm meta-analysis (Figure 1).

Table 1 summarizes the study group, design and numbers of participants of enrolled studies. Table 2 demonstrates the intervention detail of each study and their results. Both Martin et al. and Jones et al. enrolled only one participant; therefore, the studies were excluded. Five studies combined IMT with expiratory muscle training. The other five studies performed isolated IMT, and only Jones et al. [31] compared the findings in the intervention group with those in a sham group. Four studies used loads starting at 60% of MIP [14,15,32,33], three studies used loads starting at 30% of MIP [34,36,37], and one study used loads starting at 6 cm H_2_O L/s [35]. Jones et al. used an initial load of 50% MIP in the intervention group and a fixed load of 15% MIP in the sham group [31]. The intervention duration was 96 weeks in one study [36], 15 weeks in one study [35], 12 weeks in five studies [14,15,31,32], 8 weeks in one study [37], and 6 weeks in one study [34]. One study used IMT in combination with diaphragmatic gene therapy [40]. The load was 50% of MIP, and the intervention duration was 12 weeks.

### 3.2. Quality Assessment

Among the single-arm studies, one included only one participant [15], two included two participants [14,33], three included eight participants [32,36,37], one included nine participants [40], and one included 11 participants [34]. The NIH quality assessment tool for before-after (pre-post) studies indicated that seven and one studies were of good and fair quality, respectively (Table 3).

We employed funnel plots to evaluate potential publication bias in MIP, MEP, and 6MWT reporting (Supplementary Data S3–S5).

### 3.3. Effects of Intervention

#### 3.3.1. Maximal Inspiratory Pressure

**Figure 2:**
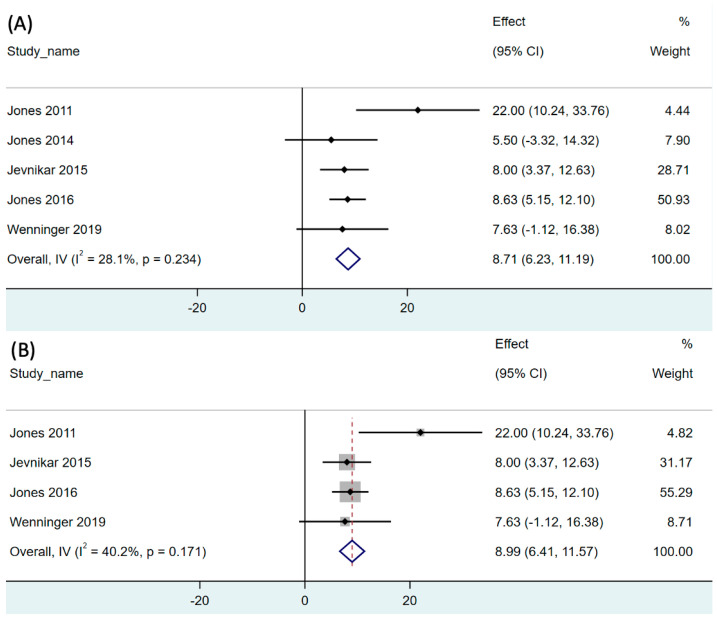
Forest plot of net change of maximal inspiratory pressure after respiratory muscle training in (**A**) both LOPD and IOPD, and (**B**) LOPD. 95% CI, 95% confidence interval. The black dot represents the point estimate and the rhombus indicates the pooled effect size.

The effect of RMT on inspiratory muscle strength in both LOPD and IOPD was reported in all included studies [14,15,31,32,33,34,36,37]. The pooled single-arm meta-analysis of five studies (31 participants) revealed that RMT significantly increased MIP by 8.71 cmH_2_O compared to that at baseline (95% CI, 6.23–11.19, *p* < 0.001). The analysis revealed moderate degree of heterogeneity (I^2^ = 28.1%, *p* = 0.234; Figure 2A).

We additionally conducted a subgroup analysis of the LOPD studies [32,33,34,36]. The pooled single-arm meta-analysis of five studies (29 participants) revealed that RMT significantly increased the MIP to 8.99 cmH_2_O compared to that at baseline in patients with LOPD (95% CI, 6.41–11.57, *p* < 0.001). The analysis revealed moderate degree of heterogeneity (I^2^ = 40.2%, *p* = 0.171; Figure 2B).

#### 3.3.2. Maximal Expiratory Pressure

**Figure 3:**
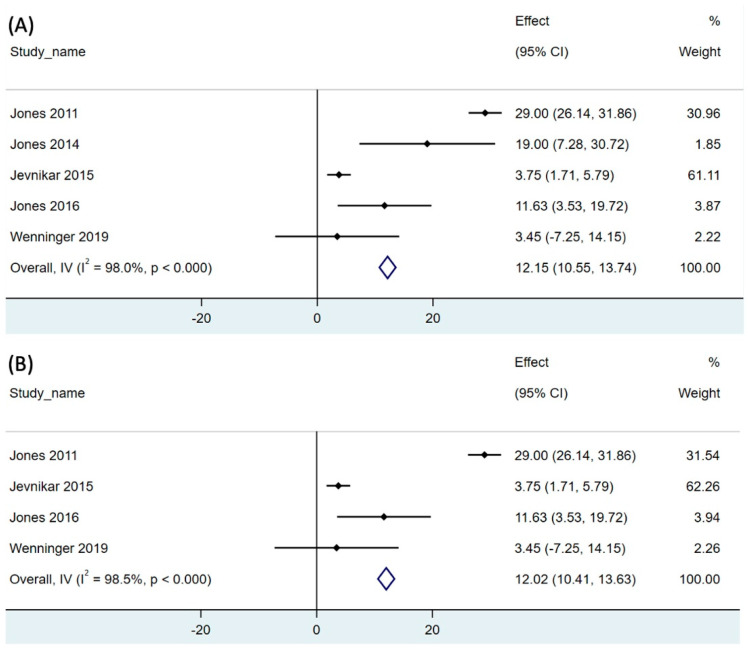
Forest plot of net change of maximal expiratory pressure after respiratory muscle training in (**A**) both LOPD and IOPD, and (**B**) LOPD. 95% CI, 95% confidence interval. The black dot represents the point estimate and the rhombus indicates the pooled effect size.

The effect of RMT on expiratory muscle strength was reported in all enrolled studies [14,15,31,32,33,34,36,37]. The pooled single-arm meta-analysis of five studies (31 participants) revealed that RMT significantly increased the MEP to 12.15 cmH_2_O compared to that at baseline (95% CI, 10.55–13.74, *p* < 0.001; Figure 3A). The analysis revealed high degree of heterogeneity (I^2^ = 98%; *p* < 0.001; Figure 3A).

We additionally conducted a subgroup analysis of the LOPD studies [31,32,33,34,36]. The pooled single-arm meta-analysis of five studies (29 participants) revealed that RMT significantly increased the MIP to 8.83 cmH_2_O compared to that at baseline in patients with LOPD (95% CI, 10.41–13.63, *p* < 0.001; Figure 3B). High degree of heterogeneity of the meta-analysis was noted. (I^2^ = 98.5%, *p* < 0.001; Figure 3B).

#### 3.3.3. Pulmonary Function: Peak Cough Flow

Three studies reported the effect of RMT on peak cough flow in Pompe disease patients [31,32,37]. We did not conduct pool analysis because of insufficient data from Aslan et al. [37] and Jones et al. [31]. Because of insufficient data, a pooled analysis was not executed. The three studies reported inconsistent results. Aslan et al. [37] and Jones et al. [31] reported insignificant change in the peak cough flow. However, in an earlier study, Jones et al. revealed an improvement in peak cough flow in three out of five patients [32].

#### 3.3.4. Pulmonary Function: Forced Expiratory Volume in 1 s and FVC

Three studies reported pulmonary function based on FVC [34,36,37]. However, due to insufficient data However, due to insufficient data from Jevnikar et al. [36] and Aslan et al. [37], a pooled analysis could not be performed. Jevnikar et al. [36] reported no detectable improvement in the predicted FVC during training. Aslan et al. [37] presented no significant changes in the pulmonary function test results, including FEV1, FVC, and FEV1/FVC. No significant changes in FVC and FEV1 were observed after respiratory muscle training (RMT) from Wenninger et al. [34].

#### 3.3.5. Functional Endurance: 6MWT

**Figure 4:**
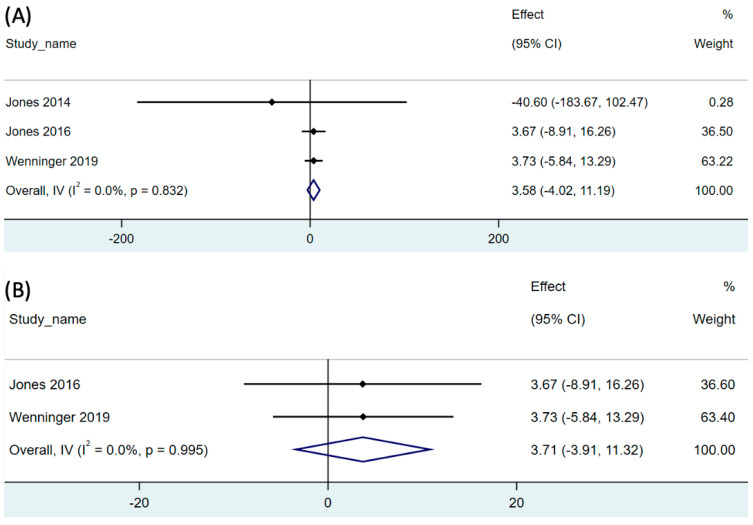
Forest plot of net change of distance of 6-min walking test after respiratory muscle training in (**A**) both LOPD and IOPD, and (**B**) LOPD. 95% CI, 95% confidence interval. The black dot represents the point estimate and the rhombus indicates the pooled effect size.

In patients with Pompe disease, functional capacity was assessed using the 6MWT. Three studies incorporated in the analysis reported the effect of RMT on the 6MWT results [14,32,34]. The combined results (21 participants) indicated insignificant differences in the 6MWT results compared to those at baseline. Specifically, an increase of 3.58 m was observed (95% CI: −4.02–11.19; *p* = 0.356; Figure 4A). The analysis revealed low degree of heterogeneity (I^2^ = 0.0%, *p* = 0.832; Figure 4A).

We additionally conducted a subgroup analysis of the LOPD studies [32,34]. The pooled single-arm meta-analysis of two studies (19 participants) revealed that RMT had no significant effect on 6MWT in patients with LOPD (95% CI: −3.91–11.32; *p* = 0.340) The analysis revealed low degree of heterogeneity (I^2^ = 0.0%, *p* = 0.995; Figure 4B).

#### 3.3.6. Functional Endurance: Borg Scale

One study incorporated the Borg Scale for Perceived Exertion into its outcomes. The average Borg scores during the training period and retraining period were 1.3–1.8 (the maximal Borg score was 5). None of the participants requested a reduction in the resistance level because of perceived exhaustion.

#### 3.3.7. Function Endurance: Gait, Stairs, Gowers, Chair

One study included Gait, Stairs, Gowers, Chair (GSGC) assessment [31]. Time to ambulate 10 m decreased by 0.5 s (SD, 1.2) and 0.7 s (SD, 1.5) in the treatment and control groups for gait assessment, respectively (*p* = 0.7666). In the Stairs assessment, the time required to climb 4 steps decreased by 0.9 s (SD, 1.0) and 0.1 s (SD, 0.9) in the treatment and control groups, respectively (*p* = 0.0346). In the Gower’s maneuver assessment, the time needed to transition from the floor to a standing position increased by 0.3 s (SD, 1.3) and 0.8 s (SD, 1.2) in the experimental and control groups, respectively (*p* = 0.2714). In the Chair subtest, the time taken to stand up from a seated position decreased by 1.1 s (SD, 2.1) and 0.2 s (SD, 1.5) in the experimental and control groups, respectively (*p* = 0.1299). The total score of GSGC test decreased by 0.8 points (SD, 1.0) and 0.1 points (SD, 1.1) in the interventional and control groups, correspondingly (*p* = 0.1499).

#### 3.3.8. QoL: Fatigue Severity Scale

One study used the Fatigue Severity Scale for analysis [31]. In the treatment group, the Fatigue Severity Scale score increased by an average of 1.7 points (SD, 5.5), whereas in the control group, it decreased by 1.3 points (SD, 3.3). These differences were statistically insignificant (*p* = 0.0984).

#### 3.3.9. QoL: Epworth Sleepiness Scale

One study used Epworth Sleepiness Scale for analysis [31]. The Epworth Sleepiness Scale score reduced by 1.2 points (SD, 2.4) in the experimental group while increased by 1.1 points (SD, 2.0) in the control group. The result revealed a statistically significant difference (*p* = 0.0160).

#### 3.3.10. QoL: Pittsburgh Sleep Quality Index

Two studies incorporated the Pittsburgh Sleep Quality index (PSQI) into their outcomes [31,37]. However, owing to insufficient data, a pooled analysis could not be performed. Aslan et al. reported no difference in PSQI scores before and after RMT. In the randomized controlled trial by Jones et al., the PSQI score decreased from 4.6 to 0.3 in the intervention group and from 2.3 to 0.7 in the sham group (*p* = 0.8677).

#### 3.3.11. QoL: Nottingham Health Profile

One study (eight patients) incorporated the Nottingham Health Profile into its outcomes [37]. Except for social isolation, the scores did not change after IMT.

### 3.4. Adverse Events

Three studies reported adverse events during the trial period [31,32,34]. In one study, one patient experienced greater-than-mild thoracic pain during RMT [32]. Wenninger et al. reported seven adverse events, with one severe event [34]. Although the patient had a left cerebral stroke in Broca’s area, it was classified as a non-related adverse event due to preexisting cerebral angiopathy. The other six adverse events were upper respiratory tract infections, myalgia of the facial and lower-back muscles, and headache in two, three, and one patient, respectively. Only three myalgia events were related to the intervention and were classified as mild-to-moderate. Jones et al. documented 21 adverse events, of which, 20 and 1 occurred in the treatment and control groups, respectively [31]. Among the adverse events, 2, 9, and 10 were classified as severe, moderate, and mild, respectively. Notably, no participant discontinued the intervention because of adverse events.

### 3.5. Publication Bias

#### 3.5.1. Maximal Inspiratory Pressure

Regarding MIP after RMT, there was no publication bias by using Egger’s test (*p* = 0.521). In addition, Egger’s test for the LOPD subgroup revealed no significant publication bias (*p* = 0.228). The corresponding funnel plots for LOPD and IOPD are shown in Supplementary Data S3(A). The funnel plot of the LOPD subgroup is shown in Supplementary Data S3(B).

#### 3.5.2. Maximal Expiratory Pressure

Regarding MEP after RMT, the result of Egger’s test (*p* = 0.987) suggested no publication bias. In addition, Egger’s test for the LOPD subgroup revealed no significant publication bias (*p* = 0.840). The corresponding funnel plots for LOPD and IOPD are shown in Supplementary Data S4(A). The funnel plot of the LOPD subgroup is shown in Supplementary Data S4(B).

#### 3.5.3. Six-Minute Walk Test

Regarding the 6MWT results after RMT, the Egger’s test did not reveal significant publication bias (*p* = 0.159). The corresponding funnel plots for LOPD and IOPD are shown in Supplementary Data S5(A). The funnel plot of the LOPD subgroup is presented in Supplementary Data S5(B).

## 4. Discussion

### 4.1. Effect of RMT on Respiratory Muscle Function

Respiratory muscle weakness has been reported in both variants of Pompe disease, that is, IOPD [7] and LOPD [17]. Based on the statement from American Thoracic Society/European Respiratory Society on pulmonary rehabilitation, IMT is recommended for patients with respiratory muscle weakness [41]. MIP and MEP are overall indicators for the strength of respiratory muscles. MIP represents the highest pressure generated during maximal inspiration, while MEP represents the greatest pressure produced during maximal expiration. In our meta-analysis, RMT significantly improved MIP and MEP. The extent of improvement in MEP seemed to be greater than that in MIP. These results are consistent with previous meta-analysis on RMT in patients with neuromuscular diseases, which showed improvements of 8.52 cmH_2_O (range, 5.22–11.83) and 12.44 cmH_2_O (range, 6.608–18.28) in MIP and MEP, respectively [42].

Although IMT does not apply a load to the expiratory muscles, it significantly increases the MEP in many diseases such as chronic kidney disease [43], heart failure [44], and pulmonary hypertension [45,46]. This may indicate that stronger inspiratory muscles can expand the thorax more effectively in preparation for MEP measurement maneuvers. This expanded position could lead to greater elastic recoil of the chest wall and lungs, potentially enhancing MEP [43]. Because RMT incorporates both inspiratory and expiratory muscle training, the additive effect may have a greater influence on MEP.

### 4.2. Effect of RMT on the 6MWT Results

The 6MWT is a submaximal exercise test used to assess aerobic capacity and endurance. The distance walked during the test serves as the outcome measurement for performance capacity. The 6MWT is frequently used to investigate the functional endurance of patients with Pompe disease. Our pooled analysis revealed no significant difference before and after RMT in all patients with Pompe disease and in those with LOPD [14,32,34]. This can be explained by several factors, first, the 6MWT primarily measures overall functional capacity, which is influenced by multiple factors, such as cardiovascular, musculoskeletal, and respiratory functions [47]. While RMT specifically targets respiratory muscles, the lack of significant improvement in walking distance may suggest that isolated respiratory muscle gains do not necessarily translate into measurable functional improvements in functional tasks like ambulation. Another consideration is the possible variability in the baseline functional capacity of the participants. Individuals with higher baseline performance on the 6MWT may have limited room for improvement, potentially diluting the effects of RMT in the overall analysis. Moreover, the duration and intensity of RMT interventions may not have been sufficient to induce significant functional changes, especially if the training period was relatively short or the intensity of the exercises was not optimally tailored to the individuals’ needs. Specifically in neuromuscular disorder, the usefulness of the 6MWT is questionable owing to confounding factors, including encompassing factors such as enhancements in respiratory muscle strength, developmental maturation, advancements resulting from continuous physical therapy, adaptations in mobility aids, and potentially, the level of effort exerted by the participant.

### 4.3. Effect of RMT on Functional Capacity and QoL

Because of the heterogeneous questionnaires used in individual studies [31,37], data synthesis was not feasible. Regarding functional capacity and QoL, the Borg, GSGC, and Fatigue Severity Scale scores and Nottingham health profile, revealed no significant improvement in all enrolled studies. However, a trend toward improvement in sleep quality was observed in the included studies. Only one study (Jones et al.) collected polysomnography data from patients with Pompe disease [31]. However, this study did not reveal significant differences between the RMT and sham groups. Therefore, functional capacity may not be significantly different owing to multifactorial issues [47]. Moreover, owing to interference from the nocturnal breathing support, obtaining meaningful polysomnography data was challenging.

### 4.4. Mechanism

RMT influences the respiratory physiology in Pompe disease via a multifaceted mechanism. RMT consists of repeated voluntary contractions of the inspiratory and/or expiratory muscles. This is achieved using pressure threshold devices with an appropriate physiological load [48,49]. Additionally, thoracic exercises enhance the mobility and expansion of the sternocostal joints, helping to prevent calcification and minimize muscle hypoextensibility or contracture [36]. Moreover, adaptations to strength training necessitate capacity to enhance efferent neural drive and elicit a proper motor response from the engaged muscle group [40]. Larger number double-blind randomized controlled trials are needed to clarify the effects of RMT in patients with LOPD.

### 4.5. RMT Intensity

Three studies initiated training at a load equal to 30% of MIP [34,36,37], four began at 60% of MIP [14,15,32,33], and one commenced at 70% of MIP [31]. According to a large meta-analysis of IMT in patients with chronic obstructive pulmonary disease, the minimal training threshold is 30% of MIP [50]. In addition to the training load, the total training intensity and amount must be considered. Jones et al. adopted training with 4500 repetitions of the IMT and EMT, in their study series [14,31,32]. Other studies calculated the total time of RMT per day to represent the training amount. The advantages of increased training in strength-training regimens are widely acknowledged. Topin et al. suggested that the effectiveness of RMT depends on the training dosage, encompassing pressure thresholds and the number of repetitions [51]. However, the optimal intensity of the RMT regimen remains unclear. Further research is warranted for comprehensively studying the effect of various training variables associated with intensity, including frequency, pressure thresholds, number of repetitions, and overall duration of the intervention. These variables can be adjusted to tailor the RMT outcomes.

### 4.6. Adherence to RMT

Adherence to RMT is substantial in patients with Pompe disease because of impaired activities of daily living (ADL) and comorbidities that hinder their training program. In our systematic review, three of the eight studies reported excellent adherence [31,32,34], with IMT completion rates of 107% and 99% [32], an EMT completion rate of 101% [32], and an RMT completion rate of 98% [31]. In the only randomized controlled trial included in this study [31], the completion rate in the sham group was 97%. Wenninger et al. observed a decrease in adherence throughout their study, with a 13% decline in the completion rate (from 107% to 94%) during the training period [34]. The program included six weeks of detraining, followed by an optional 40-week training period.

### 4.7. RMT Safety

Respiratory interventions for Pompe disease were generally safe, without adverse events reported in five of the eight studies that included safety information [14,15,33,37,40]. Three studies reported adverse events, most of which were classified as mild or moderate or were irrelevant to the training [31,32,34]. These data further support the idea that supervised RMT is a well-tolerated and safe intervention for LOPD patients.

### 4.8. Limitations and Future Outlook

Because Pompe disease is rare, the present meta-analysis included only single-arm pre- and post-intervention comparisons. Even the only study using double-blind randomized controlled trial included in the systematic review recruited 22 participants, which is considered small sample size. The small sample size potentially raises the bias.

In addition, the level of evidence for most results was either low or very low by its study design. This implies that any assessment of the outcome’s influence is highly uncertain, and there is a strong probability that new research findings will affect the confidence in determining the actual impact. Moreover, there was significant variability in the study designs across the included studies. Differences in RMT protocols, including variations in training intensity, duration, frequency, and participant characteristics, may have contributed to the heterogeneity of the results. Standardized RMT protocols and more uniform inclusion criteria in future studies would help clarify the potential benefits of RMT and allow for more precise comparisons across studies. Lastly, inconsistencies in outcome measures used in different studies may have influenced the ability to pool the data effectively. While some studies used common measures such as the 6MWT, others employed different or less established methods to assess functional capacity. This variation in outcome measures adds complexity to the interpretation of pooled results, as direct comparisons may not fully capture the nuanced effects of RMT.

ERT is the established treatment which may stabilize or potentially reduce respiratory decline for patients with Pompe disease [52]. This concurrent treatment makes it difficult to attribute changes solely to RMT. Moreover, we may need to take variable response to ERT into account. The degree to which ERT improves respiratory function can vary among individuals based on factors such as disease progression, timing of ERT initiation, and patient adherence. Meanwhile, in most studies, patients had received ERT for >10 months before starting RMT [14,31,32,33,34,36]. Therefore, the observed improvements can be attributed to RMT alone.

Finally, QoL and ADL outcomes were relatively poor in the selected studies. Although respiratory muscle function was directly evaluated, the effect of RMT on ADLs remains unknown. Because Pompe disease imposes a significant burden on patients from a humanistic perspective, including ADL evaluations in future studies is crucial [53].

Future studies focus on identifying the optimal duration, frequency, and intensity of RMT for different patient populations and explore which subgroups benefit the most, is beneficial in analyzing the therapeutic effect of RMT. Additionally, understanding the long-term effects of RMT on functional capacity and respiratory muscle strength is essential. To enhance study reliability, future research should include larger, more homogeneous samples, standardized RMT protocols, and consistent outcome measures. Longer follow-up periods would help assess the sustained benefits of RMT. Furthermore, research should explore combining RMT with other rehabilitation strategies, such as strength or endurance training of non-respiratory muscles, and investigate whether personalized RMT programs yield better outcomes than generalized approaches.

## 5. Conclusions

The findings of this study indicate that RMT significantly improves both inspiratory and expiratory muscle strength, particularly in patients with LOPD. Nevertheless, no significant improvement was seen regarding 6MWT after RMT. The effects on functional endurance, lung function, and QoL in patients with Pompe disease remain unclear. RMT could be considered for cardiac and pulmonary rehabilitation in Pompe disease patients. However, the efficacy and optimal protocol of RMT in patients with Pompe disease needs to be verified in large randomized controlled trials.

## Figures and Tables

**Table 1:** Summary of the retrieved studies investigating respiratory muscle training in Pompe disease.

Study	Types of Disease	Study Design	Number of Participants (Age)
Jones et al., 2011 [33]	LOPD	Non-randomized uncontrolled intervention trial	2 (55 and 64)
Jones et al., 2014 [14]	IOPD	Prospective, unblended, single-arm pilot study in A-B-A design	2 (5.75 and 6.5)
Jevnikar et al., 2015 [36]	LOPD	Non-randomized uncontrolled intervention trial	8 (13–58)
Aslan et al., 2016 [37]	LOPD	Non-randomized uncontrolled intervention trial	8 (23–64)
Jones et al., 2016 [32]	LOPD	Non-randomized uncontrolled intervention trial	8 (49.3 ± 8.4)
Wenninger et al., 2019 [34]	LOPD	Prospective, unblended, single-arm pilot study in A-B-A design	11 (50 ± 15.6 years)
Jones et al., 2020 [31]	LOPD	Double-blinded randomized control trial	22 RMT: 12 (53.2 ± 12.7 years) Sham: 10 (46.6 ± 13.9 years)

**Table 2:** Summary of intervention details and results of respiratory muscle training in Pompe disease.

Study	Duration (Weeks)	Frequency (Days/ Week)	Training Protocol	Intensity (%MIP or MEP)	Outcomes		
					Respiratory Muscle Strength	Lung Function	Functional Endurance and Health-Related Quality of Life
Jones et al., 2011 [33]	16–32	6	Initial 4 to 10 week: 2 × 25 rounds of IMT or EMT per day. Followed by EMT with IMT	≥60	MIP: 65% increase (+18.0 cmH_2_O) MEP: 39% increase (+17.0 cmH_2_O)	FVC: No significant change	
Jones et al., 2014 [14]	12	5	3 × 25 rounds for both IMT and EMT per day	60–70	MIP: 25.5% increase (+5.5 cmH_2_O) MEP: 50% increase (+19.0 cmH_2_O)	FVC: 8% increase (+0.85) FEV1: 5.5% increase (+0.045) PCF: 20% increase (+0.59 L/sec)	6MWT: 30.8% decrease (−40.5 m)
Jevnikar et al., 2015 [36]	96	7	Training cycle: 30% MIP for 1 min + deep slow inhalation (15 rounds) for 2 min. Total 45 min per day	30	MEP: No significant change MIP: 18% increase (+5.6 cmH_2_O)	FVC: No significant change	Gardner-Medwin- Walton scale: No significant change
Aslan et al., 2016 [37]	8	5–7	15 min, twice per day Dose:80 sets each patient	Initially 30, then 2 cmH_2_O increased per week	MEP: No significant change MIP: 30% increase (+9.0 cmH_2_O)	PCF, FVC, FEV1: No significant change	Sleep Quality: No significant change Social isolation scores: -Quality of life: Improved -Other Scores: No significant change
Jones et al., 2016 [32]	12	5	3 × 25 times of IMT and EMT per day Dose: IMT and EMT: 4500 times/each	60–70	MEP: 16% increase (+11.6 cmH_2_O) MIP: 20% increase (+8.6 cmH_2_O)	PCF: 12% increased	6MWT: 2.2% increase in distance Supine to stand: 13% time reduction 10 m walk: 3% decrease Stair climbing: 15% decrease
Wenninger et al., 2019 [34]	6	5	IMT: 105 inhalations in 30 min (divided in 7 interval)	30–40 at first then extra increase optionally by 10–15	MEP: No significant change MIP: 16% increase (+7.6 cmH_2_O)	FVC and FEV1: No significant change Capillary capnometry: No significant change	6MWT, quality of life (SGRQ, MMRC-Dysnea scale): No significant change
Jones et al., 2020 [31]	12	5	EMT + IMT: 75 times per day.	RMT: 50–70 Sham: 15	MEP: No significant MIP: No significant change change Diaphragm Thickness: No significant change	PCF: No significant change Polysomnography: no significant change	Daytime sleepiness: Significant decreased Time to climb 4 steps: Significant decreased Gross motor function and patient-reported outcomes: No significant change

6MWT: 6-min walking test; EMT: expiratory muscle training; FEV1: forced expiratory volume in the 1 s; FVC: forced vital capacity; IMT: inspiratory muscle training; MEP: maximal expiratory pressure; MIP: maximal inspiratory pressure; PCF: peak cough flow.

**Table 3:** NIH quality assessment tool for before-after (pre-post) studies with no control group: study summary.

Study	Q1	Q2	Q3	Q4	Q5	Q6	Q7	Q8	Q9	Q10	Q11	Q12	Total Score	Quality Rating
Jones et al., 2011 [33]	Y	NR	Y	NR	N	Y	Y	NR	Y	Y	NR	NA	6(11)	fair
Jones et al., 2014 [14]	Y	NR	Y	NR	N	Y	Y	Y	Y	Y	NR	NA	7(11)	good
Jevnikar et al., 2015 [36]	Y	Y	Y	Y	N	Y	Y	NR	Y	Y	NR	NA	8(11)	good
Aslan et al., 2016 [37]	Y	Y	Y	Y	N	Y	Y	NR	Y	Y	NR	NA	8(11)	good
Jones et al., 2016 [32]	Y	Y	Y	Y	N	Y	Y	NR	Y	Y	NR	NA	7(11)	good
Smith et al., 2016 [40]	Y	NR	Y	NR	N	Y	Y	NR	Y	Y	Y	NA	7(11)	good
Wenninger et al., 2019 [34]	Y	Y	Y	Y	N	Y	Y	NR	Y	Y	Y	NA	8(11)	good
Crisp et al., 2020 [15]	Y	Y	Y	Y	N	Y	Y	NR	Y	Y	Y	NA	8(11)	good

N, No; NA, Not Applicable; NR, Not Reported; NIH, National Institutes of Health; Q1, Question 1; Y, Yes.

## Data Availability

All underlying data have been presented in this article.

## Supplementary Materials

The following supporting information can be downloaded at: https://www.mdpi.com/article/10.3390/children11101209/s1, Supplementary Data S1: Search strategy for PubMed; Supplementary Data S2: Formula for Standard Deviation of Change; Supplementary Data S3: Funnel plot for the comparisons of the standardized mean difference at maximal inspiratory pressure; Supplementary Data S4: Funnel plot for the comparisons of the standardized mean difference at maximal expiratory pressure; Supplementary Data S5: Funnel plot for the comparisons of the standardized mean difference at six-minute walk test
