# Supplementary material for: Effectiveness of Respiratory Muscle Training in Pompe Disease: A Systematic Review and Meta-Analysis
Source: Children (Basel). 2024 Sep 30;11(10):1209. doi: 10.3390/children11101209 (PMC11505692; doi:10.3390/children11101209)
Supplement: Supplementary file 1 [file children-11-01209-s001.zip › children-3207675-supplementary.pdf]

Supplementary Materials

Supplementary Data S1 Search strategy for PubMed.

#1	respiratory muscle training [Title/Abstract]
#2	inspiratory muscle training [Title/Abstract]
#3	expiratory muscle training [Title/Abstract]
#4	breathing training [Title/Abstract]
#5	IMT [Title/Abstract]
#6	RMT [Title/Abstract]
#7	#1 OR #2 OR #3 OR #4 OR #5 OR #6
#8	Pompe disease [Title/Abstract]
#9	Glycogen Storage Disease Type II [MeSH]
#10	LOPD [Title/Abstract]
#11	IOPD [Title/Abstract]
#12	*#8 OR #9 OR #10 OR #11*
#13	*#7* AND #12

Supplementary Data S2 Formula for Standard Deviation of Change

$$SD_{E,change} = \sqrt{SD^2{}_{E,baseline} + SD^2{}_{E,final} - (2 \times Corr \times SD_{E,baseline} \times SD_{E,final})}$$

$SD_{E,baseline}$ is the standard deviation at baseline.

$SD_{E,final}$ is the standard deviation at the final measurement.

Corr is the correlation between the baseline and final measurements

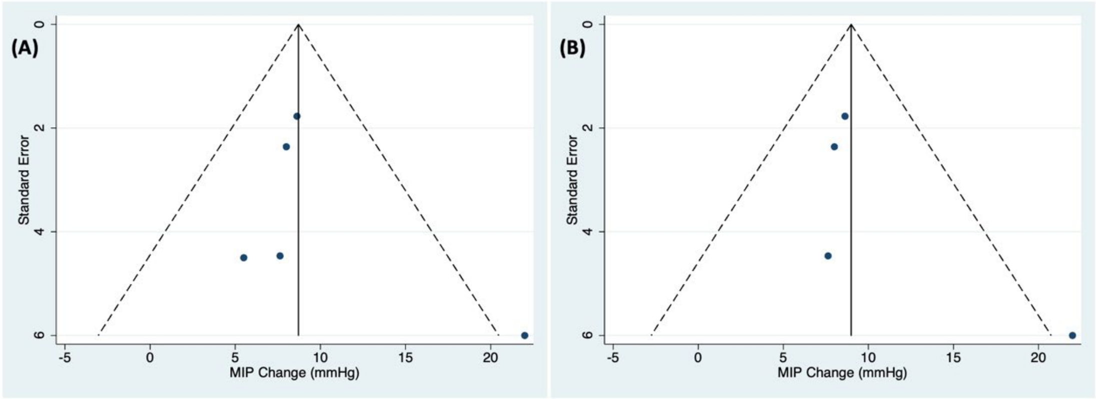

Supplementary Data S3: Funnel plot for the comparisons of the standardized mean difference at maximal inspiratory pressure of (A) LOPD and IOPD and (B) LOPD.

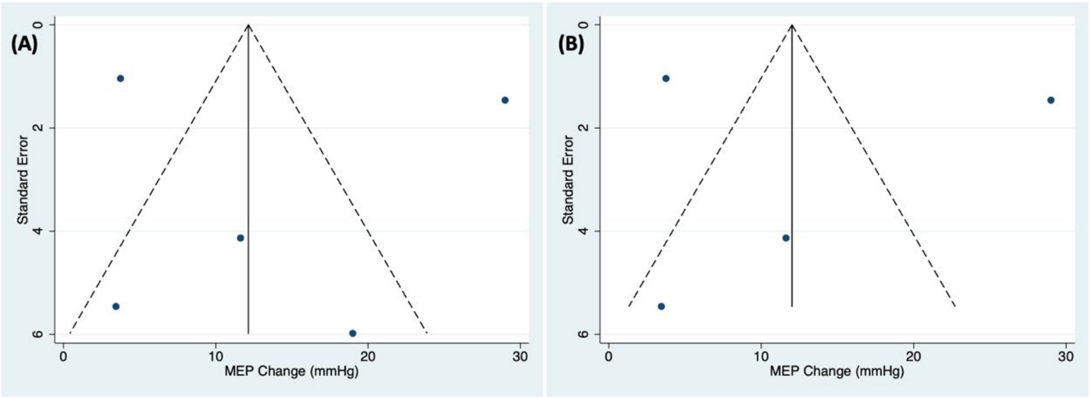

Supplementary Data S4: Funnel plot for the comparisons of the standardized mean difference at maximal expiratory pressure of (A) LOPD and IOPD and (B) LOPD.

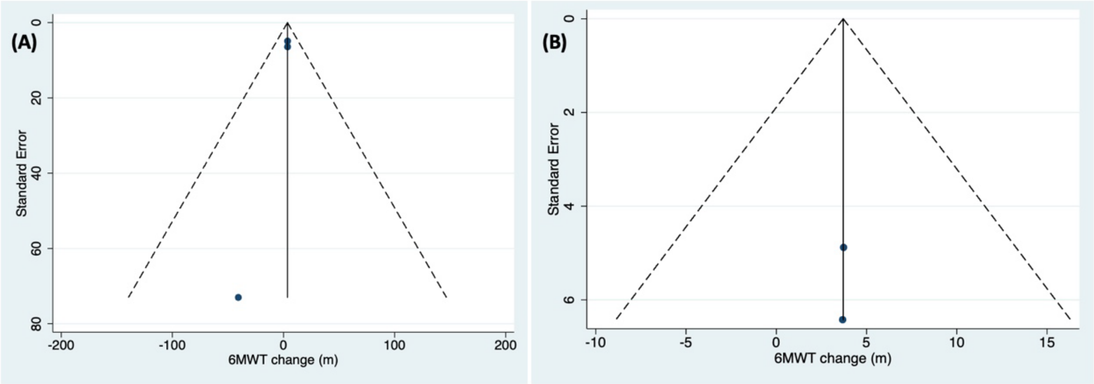

Supplementary Data S5: Funnel plot for the comparisons of the standardized mean difference at six-minute walk test of (A) LOPD and IOPD and (B) LOPD.
